# Characterizing probiotic profiles using genomic and metabolomic insights into chicken cecum-derived *Bacillus subtilis YB-1*14246

**DOI:** 10.3389/fmicb.2025.1706551

**Published:** 2025-11-13

**Authors:** Jiajun Yang, Ying Li, Jing Wang, Bo Zhang, Hao Zhang

**Affiliations:** 1 School of Animal Husbandry and Veterinary Medicine, Jiangsu Vocational College of Agriculture and Forestry, Jurong, Jiangsu, China; 2 Beijing Key Laboratory for Animal Genetic Improvement, College of Animal Science and Technology, China Agricultural University, Beijing, China; 3 Animal Husbandry and Fishery Service Center of Baoting, Hainan, China

**Keywords:** *Bacillus subtilis*, genomics, metabolism, laying performance, interplay

## Abstract

**Introduction:**

The present study aimed to explore the genetic profile and annotate the metabolic profile of chicken cecum-derived *B. subtilis YB-1*14246, which has been used in laying hens.

**Methods:**

The complete genome of *B. subtilis YB-1*14246 was analyzed and its probiotic metabolic profile was identified using genomic and metabolomic assays.

**Results and discussion:**

*B. subtilis YB-1*14246 possessed a chromosome containing 4,673,022 bp with a G+C content of 55.03%. The complete genetic sequence indicated that > 40% of the genes were coding sequences related to nutritional transportation and metabolism. *B. subtilis YB-1*14246 harboring more unique genetic clusters played a role in the metabolism of macro molecular substances and synthesized more secondary metabolites. These genes are involved in glycan metabolism, amino acid and vitamin biosynthesis, and they also contribute to nutrient absorption and utilization in chickens. In this study, nonspecific virulence factor-related genes were identified in the genome of *B. subtilis YB-1*14246. The metabolic profiles indicated that the primary functional substances were amino acids, phospholipids, eicosanoids, nucleotides, polyketides, and non-ribosomal peptides. The activities of digestive enzymes cellulase, amylase, protease, and lipase in fermented liquid of *B. subtilis YB-1*14246 were 33.49, 1.23, 9.01, and 0.49 U/mL respectively. Correlation analysis between laying performance and metabolites showed that riboflavin, p-acetaminobenzoic acid, and 6-hydroxyhexanoic acid exhibited promoting effects. Riboflavin synthesized and secreted by *B. subtilis YB-1*14246 reached 6.76 μg/L. The synthesis pathway predicted using the Kyoto Encyclopedia of Genes and Genomes (KEGG) database originates from the purine metabolism and is involved in the pentose phosphate pathway in ribosomes. The utilization and recycling patterns of riboflavin were modeled, revealing a reciprocal relationship with the host.

**Conclusion:**

Our findings revealed that the abundant nutritional genes and the production of functional metabolites by *B. subtilis YB-1*14246 supplementation may enhance the laying performance of aged hens by improving digestive abilities.

## Introduction

Probiotics are microorganisms that have beneficial effects on the host when supplemented at certain doses (Gibson et al., 2017). *Bacillus subtilis,* a probiotic strain, has been widely used as a directly fed additive in both humans and animals, with a myriad of benefits (Leistikow et al., 2024). It can optimize gastrointestinal bacterial composition, improve immune and antioxidant capacities, and enhance growth and laying performance (Liu et al., 2024; Pandey et al., 2015). Some species of *Bacillus* can survive in the guts of humans and animals, germinate, and colonize certain regions of the intestine, and function in reciprocal roles with the host (Xiao et al., 2024; Bahaddad et al., 2023; Chen et al., 2023). *B. subtilis* and *B. licheniformis* are two species commonly used as probiotic bacteria, both in humans and livestock breeding.

However, some *Bacillus* species do *not* play a promotional role in the tested animals (Sreenadh et al., 2022). The genetic profiles have been determined, and the virulence-associated genes are retained in the wild strains. Metabolic studies on species *B. alvei*, *B. cereus*, *B. brevis*, *B. coagulans*, and *B. circulans* have revealed features unfavorable to animals, such as toxicity, metabolic activity, and intrinsic antibiotic resistance, which prohibit their use (Soares et al., 2019; Wan et al., 2019). These unfavorable profiles lead to antibiotic resistance, reduced beneficial gut bacteria, and dysbiosis; therefore, further studies are required to identify suitable *Bacillus* species for practical use (Mortaz et al., 2016; Alipoor et al., 2018). The secretion of digestive enzymes and functional metabolites is critical for the development of probiotics (Yang et al., 2023). Furthermore, the origin of probiotics is an important consideration (Yang et al., 2022). Notably, the use of bacteria derived from the target animal can improve efficacy after administration (Taverniti et al., 2019). These factors collectively influence the supplementary effects of probiotics.

In different breeds of animals and at certain ages, the physiological shortcomings differ. In the face of different probiotic bacteria, the strains selected are determined by the questions that need to be addressed. In caged hens, limited physical activity and prolonged high-laying performance can readily result in cage layer fatigue (Xu et al., 2018). With increasing age, the gastrointestinal microflora undergoes compositional changes, and the laying ratio gradually declines. Middle-aged hens show impaired digestion compared to young hens. In our previous study, middle-aged laying hens (420 d old) and broiler chickens were raised and analyzed.

Chicks in the control group were fed a basal diet, while those in the *B. subtilis YB-1*14246 group were also fed a basal diet. *B. subtilis YB-1*14246 was cultured, and the fermented liquid was added to the diet in the proportion of 5 (5 L of liquid was uniformly blended with 1,000 kg of feed). Live bacteria in the diet of the *B. subtilis YB-1*14246 group reached 10^6^ colony-forming units (CFU)/g. After feeding for a certain duration, the intestinal bacterial composition of hens and broiler chickens was optimized, digestive abilities were enhanced, and the laying performance of hens and the growth of broiler chickens were improved (Yang et al., 2020; Yang et al., 2021). The probiotic physiological characteristics of *B. subtilis YB-1*14246, including digestive enzyme secretion, colonization of the cecal mucus, optimization of bacterial composition, and safety, were superior to those of other *Bacillus* strains such as *B. licheniformis*, *B. cereus*, *B. brevis*, and *B. coagulans*. Therefore, the genetic and metabolic data of *B. subtilis YB-1*14246 were utilized to interpret its probiotic profile and evaluate its suitability for practical applications.

Advances in genomics and metabolism in microbiology can aid us in further exploring and elucidating the benefits of probiotic bacteria for various applications (Yang et al., 2023). In the present study, we aimed to determine the genetic and metabolic profiles of *B. subtilis YB-1*14246 and identify potential mechanisms underlying its promotion of digestion and nutrient absorption in chickens, thereby improving performance. The rationale was to develop a novel strategy for evaluating candidate probiotic bacteria for clinical use.

## Materials and methods

### *Bacillus subtilis YB-1*14246 isolation and cultivation

*Bacillus subtilis YB-1*14246 was isolated from healthy Chinese Huainan Partridge chickens. The chicken feeding, slaughter, and isolation protocols were approved and performed in accordance with the guidelines of the Institutional Animal Care and Use Committee of China. All experimental protocols used in this study, including animal husbandry and slaughter, were approved by the Institutional Animal Science and Welfare of Jiangsu Province (no. IASWJP2015070313).

Yeast extract peptone dextrose medium (YEPD) (Qingdao Haibo Biotechnology Co., China) was prepared and sterilized for use. Cecal mucous membrane samples were scraped from chickens and incubated at 85 °C for 15 min, a temperature that kills most bacteria and is commonly used for pasteurization (Lindström et al., 2001). Notably, some heat-resistant bacteria, such as *Bacillus*, can survive this temperature. Additionally, spores germinate and propagate optimally at 32–37 °C (Pang et al., 2022).

**Figure 1:**
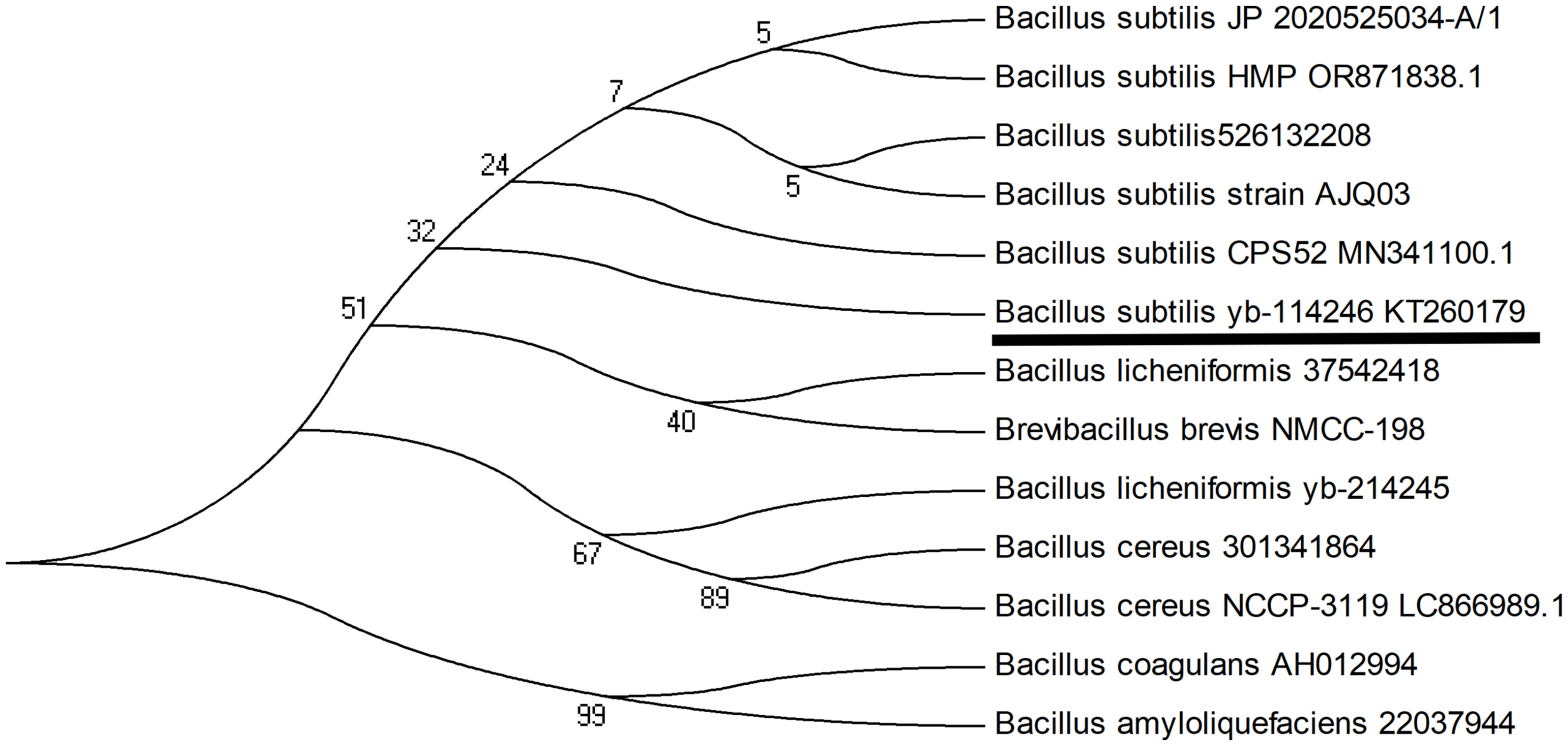
Evolutionary tree of the *B. subtilis YB-1*14246 genome compared with other *Bacillus* strains. A phylogenetic tree of *Bacillus subtilis YB-1*14246 was constructed using MEGA 4.1 software. The 16S ribosomal DNA (rDNA) of *B. subtilis YB-1*14246 was extracted, sequenced, and blasted for phylogenetic comparison with 12 strains from different sources. *B. subtilis YB-1*14246 was most closely related to *Bacillus subtilis* AJQ03 and *Bacillus subtilis* CPS52. The sequence of *B. subtilis YB-1*14246 was submitted to the NCBI and assigned the ID number for *Bacillus subtilis* strain *YB-1*14246. The total number of base pairs (bp) in the 16S rDNA was 1,466.

The samples were then streaked onto YEPD agar to isolate and culture the *Bacillus* strains. The candidate bacteria were then cultured in YEPD liquid medium (pH 7.0) at 37 °C for 18 h, and their 16S rDNA was sequenced to identify the species through comparison with other *Bacillus* strains. The *B. subtilis YB-1*14246 strain was isolated and identified. Its probiotic profile was validated in our previous study, and supplementation with 10^6^ CFU/g of live *B. subtilis YB-1*14246 in hens’ diet significantly improved laying performance (Yang et al., 2020). *B. subtilis YB-1*14246 was identified and stored at the China General Microbiological Culture Collection Center (CGMCC) under strain number CGMCC 14246. The 16S rDNA sequences of 12 *Bacillus* strains are available in a database submitted to the National Center for Biotechnology Information (NCBI). The phylogenetic tree was constructed using MEGA 4.1 (Figure 1).

### Extraction of DNA and quality analysis

The genomic DNA of *B. subtilis YB-1*14246 was isolated from cell pellets using a bacterial deoxyribonucleic acid (DNA) kit (OMEGA, D3350-00, GA, United States) according to the manufacturer’s instructions, and quality control was subsequently performed on the purified DNA samples. Agarose gel electrophoresis was performed to detect the integration of *B. subtilis YB-1*14246 DNA. The DNA fragments were purified using a magnetic bead kit (MagMAX DNA Ultra 2.0, A36570) to determine the optical density (OD260/280 = 1.8–2.0), and the DNA was weighed at >10 μg (Yang et al., 2021).

### Whole genetic sequence of *Bacillus subtilis YB-1*14246

The genomic DNA of *B. subtilis YB-1*14246 was sequenced using second- and third-generation technologies. Second-generation sequencing was performed on the Illumina HiSeq platform (PE150 mode, Shanghai BIOZERON Co., Ltd.). Genomic DNA was sheared into 350-bp fragments using a Covaris miniTUBE (Woburn, MA, United States) to construct the fragment library. Blunt ends of the fragments were generated using T4 DNA polymerase, and an “A” base was added to the 3′ end of the blunt, phosphorylated DNA fragments. The desired fragments were amplified using bridge polymerase chain reaction (PCR). The primers used were PCR 533-F “GTGCCAGCMGCCGCGGTAA” and 801-R “GGCGTGGACTTCCAGGGTATCT.” The PCR protocol consisted of an initial step at 94 °C for 5 min, followed by 35 cycles of 94 °C for 1 min, 55 °C for 1 min, and 72 °C for 1 min, with a final extension at 72 °C for 7 min. DNA clusters of *B. subtilis YB-1*14246 were then harvested. Illumina paired-end sequencing was conducted as previously described (Alhhazmi et al., 2023).

Third-generation sequencing was performed using the Pacific Biosciences platform. The entire genome of *B. subtilis YB-1*14246 was fragmented into 10-kb segments using Covaris g-TUBE (Covaris, MA), then purified, end-repaired, and ligated with SMRTbell sequencing adapters (Pacific Biosciences, CA, United States). Subsequently, a 10-kb insert whole-genome shotgun library was prepared.

The DNA was annealed, the single-stranded fragments were ligated with polymerase, and the ligation products were placed in zero-mode waveguides for sequencing on the Pacific Biosciences RS platform (California, United States) (Nemashkalo et al., 2022; Zhu et al., 2024).

### Genome assembly: functional gene annotation and virulence gene assessment

Raw data were generated using Illumina’s BaseSpace software CASAVA v1.8.2,^1^ according to the manufacturer’s instructions. Contaminated reads, including those with adapters or primers, were removed with Trimmomatic software^2^ using default parameters (seed mismatches: 2 bps, palindrome clip threshold: 30 bps, simple clip threshold: 10 bps, min adapter length: 8) (Galvanin et al., 2019). The clean data obtained from quality control were used for further analysis. Illumina data were used to evaluate genome complexity and to correct PacBio long reads.

**Figure 2:**
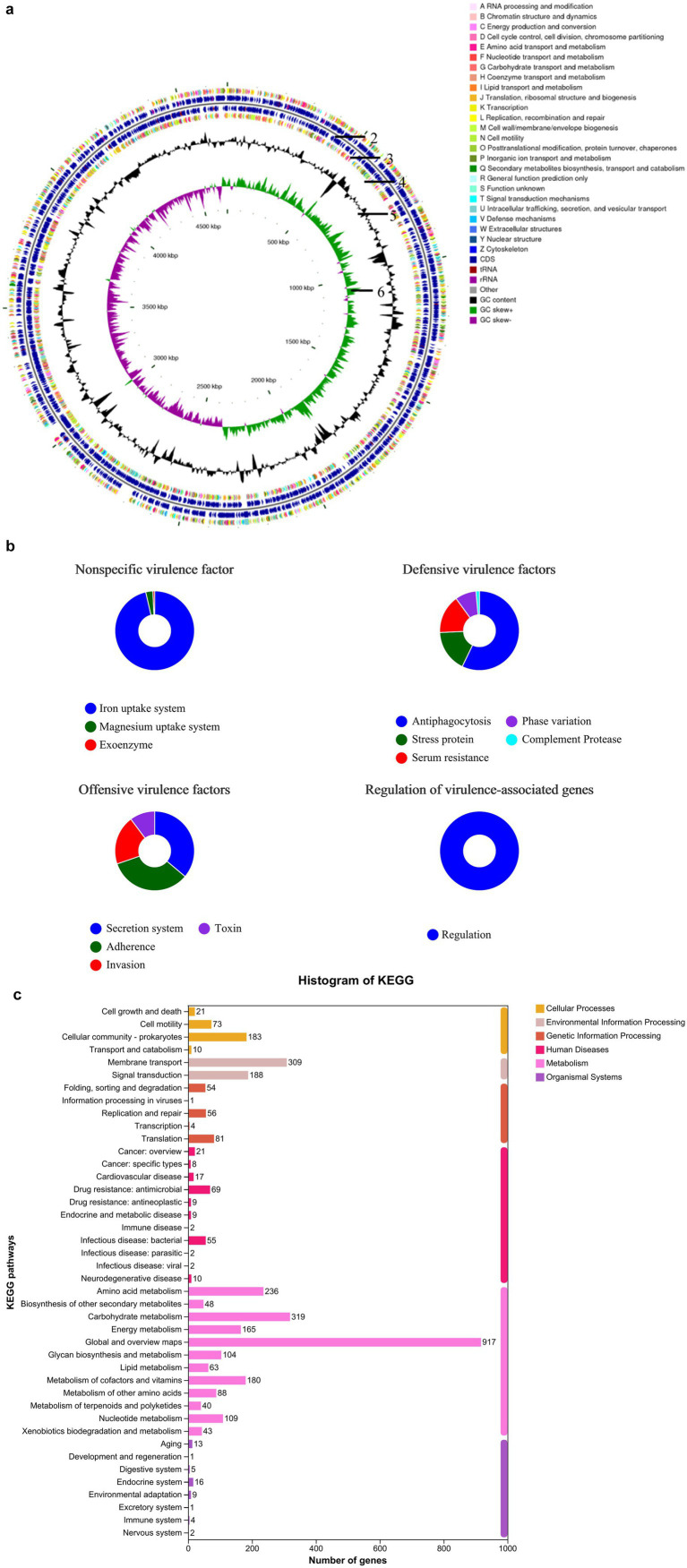
Genomic composition and functional characteristics of *B. subtilis YB-1*14246. **(a)** Genome map of *B. subtilis YB-1*14246. Analysis was performed using CGView software (version 2.0; http://wishart.biology.ualberta.ca/cgview/download.html). The first and fourth circles show the functional classification of coding sequences (CDS) on the positive and negative DNA strands, annotated with clusters of orthologous groups (COG). The second and third circles represent non-coding RNAs (ribosomal RNA [rRNA] and transfer RNA [tRNA]) embedded within the CDS on both strands. The fifth circle represents the G and C nucleotide content: outer and inner black regions indicate higher and lower G + C contents, respectively, compared to the average. The sixth circle shows GC skew values, calculated using the formula (G − C)/(G + C). Green regions indicate a positive value (G > C), and purple regions indicate a negative value (C > G). The inner region indicates the genome size. **(b)** Genetic traits of *B. subtilis YB-1*14246. When administered to the host and residing in the gut, potential risks were evaluated through genetic analysis. Although *B. subtilis YB-1*14246 contains nonspecific virulence factor-related genes, it possesses an iron uptake system that benefits host iron absorption. No regulation of virulence-associated genes was observed. To resist cellular immunity, two protective genes related to anti-phagocytosis and stress response were identified. Gene prediction indicated that *B. subtilis YB-1*14246 adheres to the intestinal mucosa upon administration, adapts to physiological conditions, and propagates continuously. **(c)** Number of functional genes in *B. subtilis YB-1*14246. Functional genes were annotated based on comparison with the Kyoto Encyclopedia of Genes and Genomes (KEGG) pathways.

For prokaryotic gene prediction, an ab initio method was used to generate gene models for *B. subtilis*. These models were identified using Glimmer 3 (Zhang et al., 2021). Subsequently, all gene models were subjected to BLAST searches against the non-redundant nucleic acid (NA in NCBI) database, SwissProt,^3^ KEGG,^4^ and Clusters of Orthologous Genes (COG^5^) to perform functional annotations using the Basic Local Alignment Search Tool for Proteins (BLASTP) module (Chen et al., 2025). The entire genome and its features are shown in the circular diagram in Figure 2. Functional genes and their classifications were annotated using BLAST and are presented in a histogram. The primary sequencing data have been deposited in the NCBI database under accession number SAMN17073208: *B. subtilis YB-1*14246 (TaxID: 1423) (NCBI SUB14634499).

### Comparative genomic analysis

To identify genes unique to *B. subtilis YB-1*14246 compared with other strains of *B. subtilis*, the complete genomes of *B. subtilis* CPS52 and *B. subtilis* AJQ03 were employed for comparative analysis. Putative housekeeping and differentiated genes among the three strains of *B. subtilis* were identified using Diamond v0.8.35^6^ and Blastp v2.3.0.^7^ A Venn diagram illustrating shared and unique genes among the three strains of *B. subtilis* was generated. Gene functions were annotated using eggNOG v4.5.1,^8^ while secondary metabolite clusters were predicted using antiSMASH v4.0.2^9^ and analyzed through KEGG pathway mapping.

### Cultivation of *Bacillus subtilis YB-1*14246 and digestive enzyme assay

*Bacillus subtilis YB-1*14246 was cultured and colonized in YEPD medium (pH 7.0) following a 1% inoculation and incubation at 37 °C for 18 h. Viable bacteria present during fermentation were quantified using the plate count method. A tenfold serial dilution was performed up to 10^−7^, and 100 μL of the diluted samples were spread on solid YEPD agar to enumerate live bacteria.

For the digestive enzyme assays, *B. subtilis YB-1*14246 was cultured in YEPD medium at 37 °C for 18 h. The fermented broth was collected and analyzed for cellulase, α-amylase, β-amylase, protease, and lipase using biochemical kits purchased from Nanjing Jiancheng Bioengineering Institute (Nanjing, China) (Yang et al., 2021). Cellulose was hydrolyzed to cellobiose or glucose, which reduces 3,5-dinitrosalicylic acid (DNS) under alkaline conditions to produce a maroon-colored amino compound. A glucose solution (2 mg/mL) was used as the standard. Absorbance was measured spectrophotometrically. Cellulase activity was calculated based on absorbance, with 1 μg of glucose hydrolyzed per minute defined as one enzyme activity unit (1 U). The substrate used to assess protease activity was casein, which is reduced to a blue compound. Notably, the solution contained 50 μg/mL casein, which served as the standard, and 1 μg of casein hydrolyzed per minute was defined as one enzyme activity unit (1 U). For the detection of lipase activity, triglyceride was used as a substrate, which was hydrolyzed by lipase, resulting in a reduction in the solution’s turbidity. One enzyme activity unit (1 μg) was defined as the enzyme required to hydrolyze 1 μg of triglyceride per minute. Starch was hydrolyzed by α-amylase and β-amylase to form α-1,4-glucanamylopectin. Notably, α-amylase and β-amylase were absorbed using horseradish peroxidase (HRP) conjugated antibodies. The detected samples and standardized concentrations of amylase-containing samples were used for incubation, and the substrate (tetramethylbenzidine) was used for chromogenesis. Tetramethylbenzidine was catalyzed, forming a blue compound. The absorbance of the solution was measured using a microplate reader at 450 nm. The YEPD medium was treated as the control. Ten replicates were used for each treatment.

### Metabolomic assay on *Bacillus subtilis YB-1*14246

*Bacillus subtilis YB-1*14246 was cultured in YEPD medium (pH 7.0) at 37 °C for 18 h. The fermentation mixture (100 μL of fermented medium with *B. subtilis YB-1*14246) was centrifuged at 3,000 rpm for 10 min to remove the supernatant. The bacterial pellet was resuspended in 100 μL of double-distilled water and washed twice via centrifugation at 4,000 rpm for 10 min. Cells were then subjected to ultrasonic disruption at 400 W, operating for 2 s with 6-s intervals, repeated for 10–20 min. This pretreatment followed the methodology described previously (Yang et al., 2023). Control samples were sterilized in YEPD medium (control). The fermented medium without *B. subtilis YB-1*14246 cells was treated as the sample of metabolites secreted out of the cells of *B. subtilis YB-1*14246 (F). Cell samples treated with ultrasonic disruption were used to detect intracellular metabolites secreted by *B. subtilis YB-1*14246 (J). Samples were vortex-mixed for 30 s and subjected to low-temperature ultrasonic extraction (4 °C, 40 kHz).

Samples were stored at −20 °C for 30 min, then centrifuged at 13,000 rpm for 30 min. The pellet was reconstituted in 120 μL of 50% acetonitrile solution and transferred into a vial for ultra-performance liquid chromatography tandem mass spectrometry (UPLC-MS) analysis (Ohtake et al., 2017).

The samples were pumped at 0.3 mL/min and injected into an Agilent Infinity Poroshell 120 ECC18 system (column 2.1 × 100 mm, 2.7 μm, 695575-902, Folsom, CA). After sample preparation, the capillary and spray nozzle voltages were set to 3,500 V and 500 V, respectively. The electron impact (EI) ion source temperature was set to 350 °C with a 90 eV electron beam. The injector temperature was set to 325 °C, with a solvent delay of 7 min. The nitrogen gas flow rate was set to 3.0 mL/min. A total of 20 μL of each sample was mixed with a quality control (QC) sample for error correction.

### Annotation of proposed probiotic mechanisms

To investigate the correlation between secreted functional metabolites and laying performance, statistical analysis was conducted using SciPy (Python v1.0.0). The biosynthetic pathway of riboflavin produced by *B. subtilis YB-1*14246 was predicted and annotated via KEGG using BLAST.

To measure the riboflavin concentration in the fermented liquid of *B. subtilis YB-1*14246, a riboflavin standard (Alladdin Company, United States) was diluted to 1, 5, 10, 50, 100, and 1,000 μg/L to create a calibration curve. Quantification was performed using the UPLC-MS protocol (Wang et al., 2023). Primary metabolism data have been deposited at the NCBI under the accession number SUB14634499. The metabolomic data for *B. subtilis YB-1*14246 are available under PRJNA1142186 and SAMN42929163: *B. subtilis* metabonomes (TaxID: 1239).

### Statistical analyses

Data from assays on digestive enzymes and levels of functional substances secreted by *B. subtilis YB-1*14246 were analyzed using one-way analysis of variance (ANOVA) with the GLM procedure in SPSS Statistics software (Version 20.0), with significance set at a *P* < 0.05. Information on identified functional genes (by COG/KEGG categories), major metabolic compounds detected and their concentrations, and digestive enzyme secretion by *B. subtilis YB-1*14246 are summarized in Supplementary Table S1.

## Results

### Phylogenetic analysis

The evolutionary relationship of *Bacillus subtilis YB-1*14246 with other *Bacillus* strains was inferred based on its 16S rDNA sequence. An evolutionary tree was constructed and is shown in Figure 1. When compared with 12 other *Bacillus* strains submitted to the NCBI nucleotide database, *B. subtilis YB-1*14246 showed close phylogenetic proximity to *B. subtilis* AJQ03, which was isolated from the intestinal tract of *A. japonica*, and *B. subtilis* CPS52 (a strain used as a probiotic supplement).

### Comparative genomic analysis

**Figure 3:**
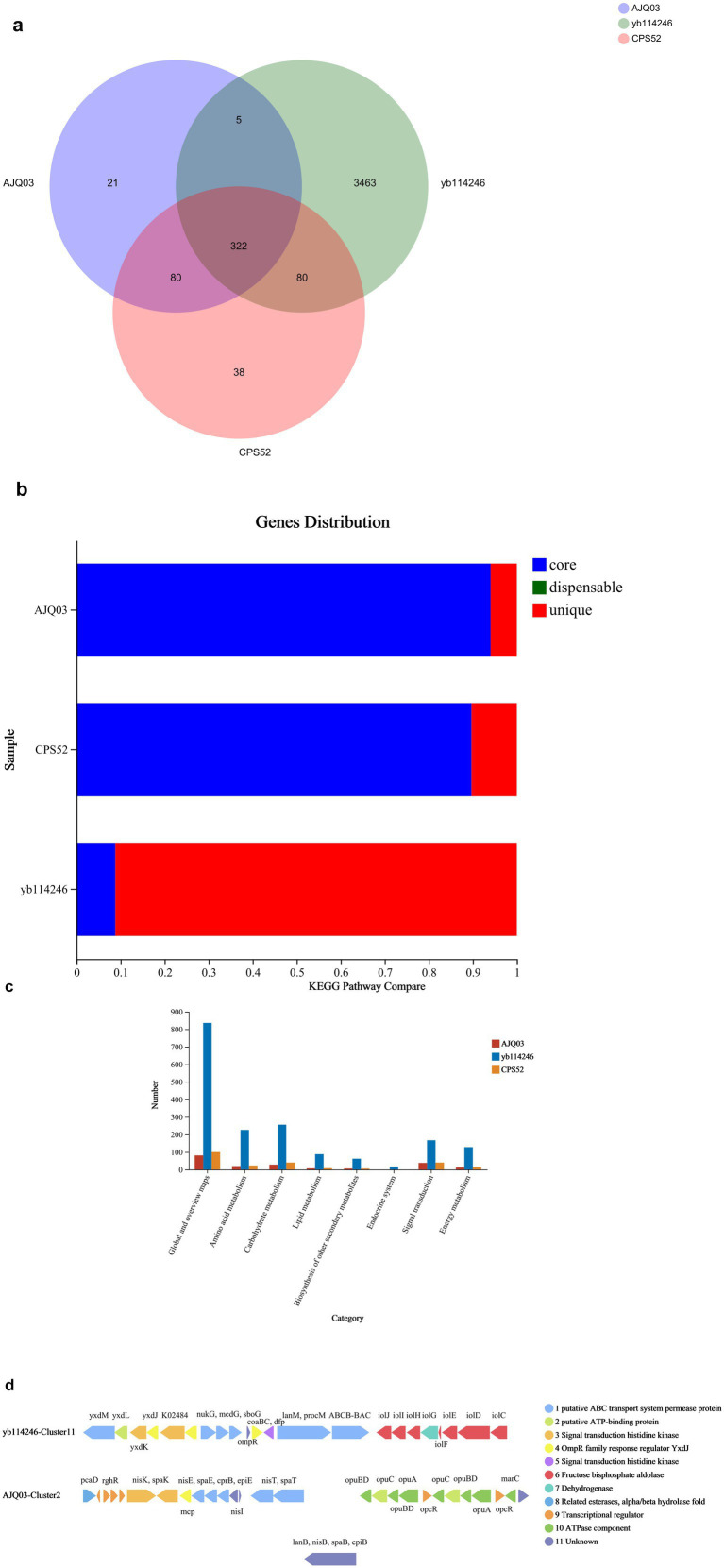
Comparative genetic information of three strains of *B. subtilis*. **(a)** Venn diagram showing shared housekeeping genes and different genes among three *B. subtilis* strains. **(b)** Comparison of core genes and unique genes among the three *B. subtilis* strains. Core housekeeping genetic clusters shared by three strains are indicated in blue; unique genetic clusters owned by different *B. subtilis* strains are indicated in red. **(c)** Functional gene comparison of three genes of *B. subtilis* was performed using KEGG. **(d)** Genetic clusters on lantipeptides synthesized by *B. subtilis YB-1*14246 and *B. subtilis* AJQ03. One line represents a genetic cluster. One arrow represents a genetic segment; the arrow length indicates the length of the genetic segment; the arrow direction indicates the sense and antisense encoding sequences; and the color represents the function.

The Venn diagram of core housekeeping genes and other genes across three *B. subtilis* strains is shown in Figure 3a. A total of 198,556 and 433,806 bps were noted for *B. subtilis* CPS52 and *B. subtilis* AJQ03, respectively, which were shorter than *Bacillus subtilis YB-1*14246. There were 322 housekeeping genetic clusters shared by the three strains. Housekeeping genetic clusters among the three strains of *B. subtilis YB-1*14246 covered 8.9% of the total of 4,880 genes, and the genes were unique genetic clusters that covered 91.1% in *B. subtilis YB-1*14246. The situations in *B. subtilis* CPS52 and *B. subtilis* AJQ03 were different. Unique genetic clusters covered <50% of the total genes, whereas housekeeping genetic clusters accounted for 61.3 and 75.1%, as shown in Figure 3b and Supplementary Table S2.

The functions of genetic clusters were predicted, and the differences are shown in Figure 3c. *B. subtilis YB-1*14246 has more genes involved in the metabolism of amino acids, carbohydrates, and lipids compared with *B. subtilis* CPS52 and *B. subtilis* AJQ03. In addition, some genetic clusters can synthesize certain types of secondary metabolites. These functional genetic clusters, owned by three strains of *B. subtilis,* synthesize secondary metabolites, including non-ribosomal peptides (NRPs), lantipeptides, terpenes, trans-AT PKS, Type III PKS, and bacteriocins, as indicated in Supplementary Table S3. In *B. subtilis YB-1*14246, all genetic clusters are involved in synthesizing six types of secondary metabolites, which is more than both *B. subtilis* CPS52 and *B. subtilis* AJQ03.

The clusters for synthesizing lantipeptides were predicted through KEGG. Both *B. subtilis YB-1*14246 and *B. subtilis* AJQ03 harbor clusters that synthesize lantipeptides, unlike *B. subtilis* CPS52. The functional genetic segments are clarified in Figure 3d. Among these 11 segments, *B. subtilis YB-1*14246 and *B. subtilis* AJQ03 each have eight genetic segments in the clusters, none of which are identical; five segments are shared between them.

### Unique genetic traits and functions

The genomic profile of *B. subtilis YB-1*14246 is shown in Figure 2a. The strain possesses a single chromosome. The combined sequencing depth from second- and third-generation technologies was 132.19×, and a total of 15,490 valid, clean reads were obtained, yielding 4,673,022 bp and covering 99.29% of the genome. The GC content was 55.03%. Coding sequences (CDS) from both the positive and negative DNA strands were identified and functionally annotated using the KEGG database. Notably, > 40% of the CDS were associated with nutrient transport and metabolism, with major categories including carbohydrate, amino acid, lipid, and nucleotide transport and metabolism, as presented in Figure 2b and Supplementary Table S1.

Nonspecific virulence factor-related genes were identified in *B. subtilis YB-1*14246. However, genes related to iron and magnesium uptake systems were present. Defense mechanisms encoded in the genome included anti-phagocytic factors, stress proteins, serum resistance proteins, phase variation elements, and complement proteases. Adherence-related genes were also detected, which may contribute to intestinal colonization upon oral supplementation. To further elucidate the beneficial functions of the genes, KEGG pathway analysis was conducted (Figure 2c). A total of 917 genes were involved in metabolic processes, including 319 in carbohydrate metabolism, 104 in glycan metabolism, 236 in amino acid metabolism, and 88 in other pathways. In addition, 109 and 63 genes were implicated in nucleotide and lipid metabolism, respectively. A total of 48 genes were annotated under the biosynthesis of secondary metabolites, including those for vitamins, short peptides, and cholesterol.

### Metabolomic profile and functional metabolites

**Figure 4:**
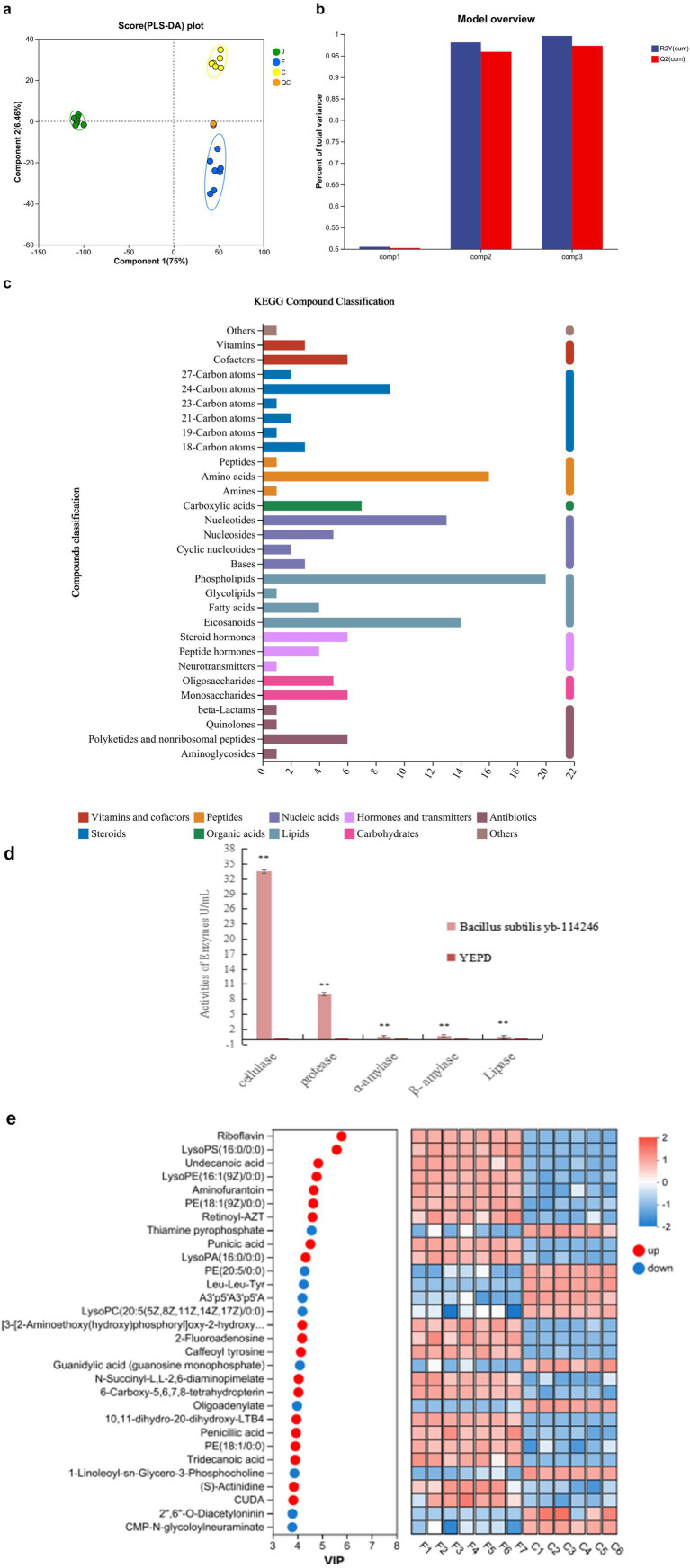
Metabolic profile and function of *B. subtilis YB-1*14246. **(a)** Analysis of metabolites from *B. subtilis YB-1*14246 cells and their fermentation compared with the culture medium. Partial least squares discriminant analysis (PLS-DA) was used to evaluate differences between treatments. YEPD medium was the control (C, *n* = 12), *B. subtilis YB-1*14246 treated with sanctions (J, *n* = 12), fermented supernatant (F, *n* = 12), QC: quality control (*n* = 12). **(b)** PLS-DA model overview. The accuracy of PLS-DA was cross-validated, and the model is shown. R2X and R2Y indicate the interpreting incidences on the X and Y matrices of the constructed model. R2X (cum) and R2Y (cum) indicate the accumulated interpreting incidences. Q2 represents the predictive capacity, and Q2 (cum) indicates the accumulated predictive capacity. The closer the R2X (cum), R2Y (cum), and Q2 (cum) values are to 1, the more reliable the model predictive capacity is. When Q2 (cum) > 0.5, the predictive capacity was good, whereas values of < 0.5 indicated unreliability. **(c)** Major metabolites secreted by *B. subtilis YB-1*14246. Compounds were classified using the KEGG database. Amino acids, phospholipids, and eicosanoids were the three primary types of macromolecules. **(d)** Activity of digestive enzymes in the fermented liquid of *B. subtilis YB-1*14246. The YEPD was the control (*n* = 10), fermented medium was the treatment, and the activities of cellulase, α-amylase, β-amylase, protease, and lipase were analyzed (*n* = 10). One-way ANOVA analysis was performed on the activities of digestive enzymes. * in the same column indicates *p* < 0.05, and ** means *p* < 0.01. **(e)** Differences in compound composition between the fermentation liquid of *B. subtilis YB-1*14246 and culture medium. Based on the PLS-DA module, the first component was used to construct the contributed values on all the metabolites, termed VIP values. The VIP values differed among the metabolites (−1 < VIP values < 1). Higher values indicate significant differences between the Control YEPD and the supernatant of *B. subtilis YB-1*14246. A total of 30 representative compounds were selected to compare the differences between the two groups.

To characterize the metabolites secreted by *B. subtilis YB-1*14246, a UPLC-MS-based analysis was performed. The metabolites in QC and treated samples were clustered together and are shown in Figure 4a. The data were evaluated using partial least squares discriminant analysis (PLS-DA). The PLS-DA was cross-validated; the model is shown in Figure 4b, and the results are presented in Supplementary Table S4. All three values of the primary component Q2 (cum) are > 0.5, indicating good reliability of the prediction. Culture media and incubation temperature were the primary variables influencing the secretion profile, collectively accounting for 81.46% of the total metabolites.

The bioactive molecular compounds were classified using the Kyoto Encyclopedia of Genes and Genomes, which showed that the major bioactive compounds included vitamins, cofactors, various carbon atoms, amino acids, peptides, nucleotides, phospholipids, eicosanoids, polyketides, and non-ribosomal peptides (Figure 4c). Among these, amino acids, phospholipids, and eicosanoids represent the largest proportions.

To evaluate the peptide bioactivity, the amino acid components were analyzed after digestion. The activities of digestive enzymes secreted into the fermentation broth of *B. subtilis YB-1*14246 were measured as follows: 33.49 U/mL cellulase, 1.23 U/mL amylase, 9.01 U/mL protease, and 0.49 U/mL lipase (Figure 4d). The viable cell count in the fermented liquid reached 7.8 × 10^10^ colony-forming units per milliliter (CFU/mL).

### Correlations between metabolites and laying performance

**Figure 5:**
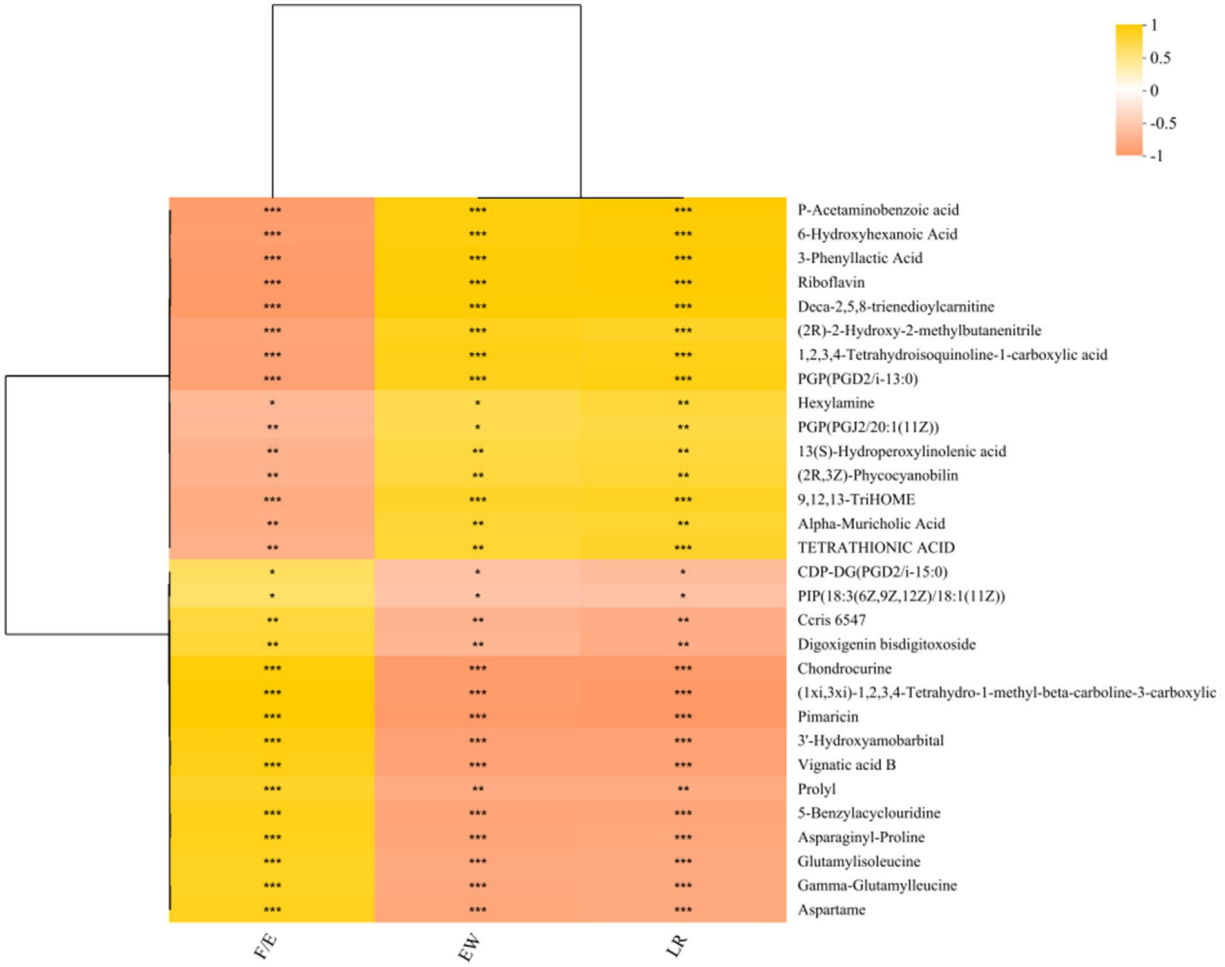
The correlation between metabolites and laying performance. In a previous study (Yang et al., 2020), hens in the control group were fed a basal diet, while hens in the *B. subtilis YB-1*14246 group were fed a diet containing *B. subtilis*. The average laying rate reached 75.90% during 72–80 weeks of age. *Bacillus subtilis YB-1*14246 was cultured in YEPD medium for 18 h at 37 °C, then the fermented liquid was harvested and added to the diet in the proportion of 5‰ (5 L of liquid was uniformly blended with 1,000 kg of feed). The average laying rate of supplemented hens reached 82.31% during the feeding time. To assess the relationship between metabolites and laying performance, Spearman’s correlation analysis was performed. Several key metabolites—P-acetaminobenzoic acid, 6-hydroxyhexanoic acid, 3-phenyllactic acid, riboflavin, and deca-2,5,8-trienedioylcarnitine—were significantly associated with laying rate (LY), egg weight (EW), and feed-to-egg weight ratio (F/E).

After cultivation, *Bacillus subtilis YB-1*14246 colonized the YEPD medium and secreted several functional metabolites. The levels of riboflavin, undecanoic acid, aminofurantoin, 5-methylthioadenosine, PE (15:0/0:0) (17:1/0:0), punicic acid, retinoyl-AZT, lysoPS (16:0/0:0), lysoPE (16:1(9Z)/0:0) (14:1(9Z)/0:0), tridecanoic acid, and penicillic acid were significantly elevated (Figure 4e). Of all the macromolecular substances, the concentration of riboflavin improved the most; the VIP value reached 2. A correlation analysis between functional substances metabolized by *B. subtilis YB-1*14246 and laying performance (Figure 5) identified *p*-acetaminobenzoic acid, 6-hydroxyhexanoic acid, 3-phenyllactic acid, riboflavin, and deca-2,5,8-trienedioylcarnitine as the most significantly associated compounds, as shown in Supplementary Table S1.

### Riboflavin synthesis, levels, and reciprocal relation with the host

**Figure 6:**
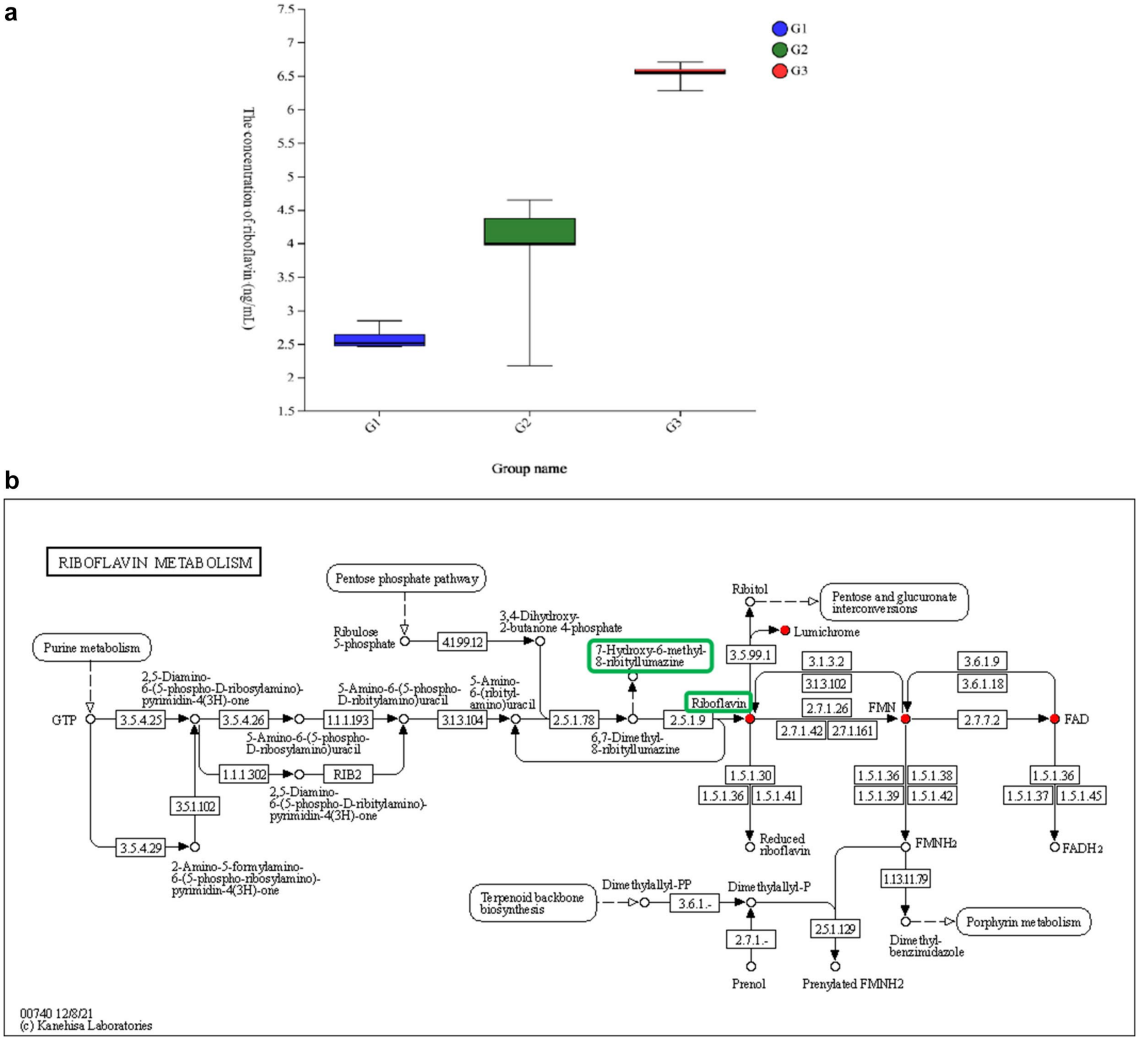
The level of riboflavin synthesized in *B. subtilis YB-1*14246 and its pathway. **(a)** The level of riboflavin metabolized by *B. subtilis YB-1*14246. G1: YEPD medium (*n* = 12); G2: riboflavin secreted from the cytoplasm into the fermented supernatant by *B. subtilis YB-1*14246 cells (*n* = 12); G3: riboflavin retained in the cytoplasm of *B. subtilis YB-1*14246 treated with sanctions (*n* = 12). **(b)** The synthesized pathway of riboflavin.

Among the 30 identified compounds, riboflavin was the only metabolite that positively influenced laying performance and was produced in large amounts by *B. subtilis YB-1*14246. Therefore, its biosynthetic pathway was annotated. The intracellular and extracellular concentrations of riboflavin in *B. subtilis YB-1*14246 were 4.15 and 6.76 μg/L, respectively (Figure 6a). Riboflavin synthesis in *B. subtilis YB-1*14246 originates from purine metabolism within the ribosomes, as illustrated in Figure 6b. The biosynthetic cascade begins with the hydrolysis of GTP to form 2,5-diamino-6-(5-phospho-D-ribosylamino)pyrimidin-4(3H)-one, which is subsequently converted into 5-amino-6-(5-phospho-D-ribosylamino)uracil, then to 5-amino-6-(ribosylamino)uracil. Upon oral administration of *B. subtilis YB-1*14246 to the ileum, riboflavin was synthesized to facilitate cell digestion in the intestinal mucosal membrane.

## Discussion

In a previous study, *Bacillus subtilis YB-1*14246, isolated from chickens, was shown to colonize and proliferate in the cecum of chicks when their diet was supplemented with 10^6^ CFU/g (Yang et al., 2020; Yang et al., 2021). Its practical use has been associated with improvements in laying performance in hens (Xiao et al., 2024). Compared to 12 other *Bacillus* strains, *B. subtilis YB-1*14246 exhibited a unique DNA sequence, distinguishing it from other homologous *B. subtilis* strains. Although *B. subtilis* AJQ03 (isolated from the intestinal tract of *Anguilla japonica*) and *B. subtilis* CPS52 have been used as probiotic supplements, *B. subtilis YB-1*14246, isolated from the chicken cecum, follows a similar pattern of isolation and application for dietary supplementation. Its ability to secrete digestive enzymes, colonize the cecum, and modulate bacterial composition contributes to enhanced production efficiency in laying hens and growth in broiler chickens. However, its full genetic and metabolic potential remains to be elucidated.

Genomic analysis has revealed that numerous CDSs in the genome of *B. subtilis YB-1*14246 are involved in nutrient transport and metabolism, more than in *B. subtilis* AJQ03 and *B. subtilis* CPS52. Some genetic clusters are unique and are involved in the metabolism of macromolecular substances. Among these clusters, certain secondary metabolites can be synthesized, such as NRPs, lantipeptides, terpenes, trans-AT PKS, Type III PKS, and bacteriocins (Mohite et al., 2025). Lantipeptides, trans-AT PKS, Type III PKS, and bacteriocins can be used as antimicrobial agents extracted from metabolites secreted by these bacteria and play important roles in biomedicine (Ueoka et al., 2022). Lantipeptides can also be used as antioxidants to alleviate heat stress (Manetsberger et al., 2025).

Further functional classification showed that many of these genes are related to the metabolism and biosynthesis of nutritional compounds. A high number of genes involved in carbohydrate, glycan, and amino acid metabolism were identified in *B. subtilis YB-1*14246. More functional genes in defined gene cluster families are possessed by *B. subtilis YB-1*14246 than by *Bacillus cereus* ATCC 14579, *Bacillus megaterium* NBRC 15308 (Grubbs et al., 2017), referred to as *Ligilactobacillus salivarius* (accession No. MH517354.1) (Yang et al., 2023), and *Bacillus subtilis* (Steinke et al., 2021; Kim et al., 2023).

Notably, dietary supplementation with *B. subtilis YB-1*14246 enables it to survive destruction by gastric acid and intestinal juices, ultimately reaching and propagating in the small intestine (Javid et al., 2023). Its functional role in feed is largely dependent on its ability to secrete a broad range of digestive enzymes capable of degrading macromolecular nutrients—not only for its own survival but also for host nutrient utilization (Pang et al., 2022). Genomic inspection confirms that *B. subtilis YB-1*14246 harbors numerous genes encoding such digestive enzymes, a property commonly exploited in poultry production, where enzyme additives are routinely used (Cao et al., 2023).

The digestive enzymes secreted by *B. subtilis YB-1*14246 include cellulase, amylase, protease, and lipase. It is speculated that the diet included digestive enzymes to support the chicken’s digestion. The supplementary dose reached 300 U/kg, such as cellulase or hydrolase. Recently, digestive enzymes produced by probiotic bacteria have been isolated and added to animal diets. Notably, some strains of *Bacillus* and *Lactobacillus* are often used (Wang et al., 2024). Fermented *B. subtilis YB-1*14246 has been supplemented at a dose of 5 L/t (5 L fermented *B. subtilis YB-1*14246 liquid in every ton of feed). The 225 U digestive enzyme dose was supplied at 1 g/kg of diet. Supplemented *B. subtilis YB-1*14246 residing in the intestine can produce additional digestive enzymes to assist intestinal digestion. These enzymes play additional roles in both laying hens and broiler chickens by aiding the host in digesting nutritional macromolecules (Salem et al., 2023). Notably, the activities of cellulase and protease possessed by *B. subtilis YB-1*14246 are higher than those reported for *Ligilactobacillus salivarius* and *Bacillus licheniformis YB-2* (Yang et al., 2023; Yang et al., 2020). Under suitable conditions, *Bacillus* spores can germinate, develop, and grow. Optimal nutrition is required for its propagation, and additional compound enzymes should be secreted to help host digestion (Bahaddad et al., 2023; Veening et al., 2008). This reciprocal relationship benefits both the host and *B. subtilis YB-1*14246.

Colonization of the gastrointestinal tract by *B. subtilis YB-1*14246 supports essential physiological functions, such as digestion and immunity. To ensure the host’s safety, virulence factors and environmental adaptability were evaluated. The genome analysis of *B. subtilis YB-1*14246 predicted that nonspecific virulence factors pose a threat to host health. Defense gene prediction identified adherence-related traits that facilitate bacterial colonization of the gut and support survival following macrophage-mediated clearance (Scriba et al., 2024). A previous study evaluated the adherence traits. A chicken diet supplemented with *Bacillus subtilis YB-1*14246 showed that colonized *Bacillus subtilis* can be detected in the cecal mucosa through fluorescence *in situ* hybridization (FISH) (Yang et al., 2021). The adherence trait is reciprocal for hosts and *Bacillus subtilis YB-1*14246. Certain uncharacterized genes in *B. subtilis YB-1*14246 may pose a risk to the host under specific conditions, such as during wound infections (Zhou et al., 2023; Rahman et al., 2022). Previous studies have also indicated that wounds and low immune status are associated with probiotic bacterial infections (Chen et al., 2025).

Genes involved in the biosynthesis of secondary metabolites, such as vitamins and peptides, contribute to improving host nutrition and immunity. Metabolic profiling revealed that *B. subtilis YB-1*14246 secretes compounds involved in the metabolism of nucleotides, carbohydrates, lipids, peptides, and amino acids. Among the distinct fermentation-derived compounds identified are vitamins, undecanoic acid, aminofurantoin, 5-methylthioadenosine, PE (15:0/0:0) (17:1/0:0), punicic acid, retinoyl-AZT, lysoPS (16:0/0:0), lysoPE (16:1(9Z)/0:0) (14:1(9Z)/0:0), tridecanoic acid, and penicillic acid. These functional metabolites play important roles in maintaining host metabolic activity and providing protection against pathogens (Vera-Santander et al., 2023; Vieco-Saiz et al., 2024).

The correlation between secreted functional compounds and egg-laying performance revealed that P-acetaminobenzoic acid, 6-hydroxyhexanoic acid, 3-phenyllactic acid, riboflavin, and deca-2,5,8-trienedioylcarnitine are notably correlated with laying performance. Among all these macromolecular substances, riboflavin concentration improved most significantly. Therefore, the synthesized pathway and levels of riboflavin should be identified. Abundant bacteria residing in the intestinal mucosa play reciprocal roles with the host. Notably, riboflavin secretion is a probiotic effect in metabolites of *B. subtilis YB-1*14246 (Nikoopour and Singh, 2014; Lindahl, 2022).

In our previous study, the supplementary dose of *Bacillus subtilis YB-1*14246 was 5 L/t in the diet (Yang et al., 2020). The total riboflavin supplementation was 0.034 mg per ton of diet. Notably, the supplementary level was notably lower than the standardized level. In addition, *B. subtilis YB-1*14246 colonized the cecum and used chyme to propagate and produce additional riboflavin, which can be used by microbiota such as *Akkermansia* and *Verrucomicrobium*. The metabolites and remaining riboflavin can be transported into the cytoplasm via ATP-binding cassette transporters, thereby improving the surveillance of intestinal immune cells and the antioxidant capacity of liver cells (Millán-García et al., 2024). Intestinal MAIT cells (mucosal-associated invariant T cells) can be induced by metabolites derived from the riboflavin biosynthetic pathway (Germain et al., 2025). When the riboflavin level in the cultivation media reached 12.76 ng/L, the antioxidant capacity of Hep G2 cells was reported to improve (Zhang et al., 2022).

The synthesized riboflavin is transported in the blood circulation (Liu et al., 2021; Riley, 2003), enters the cytoplasm, and is incorporated into the tricarboxylic acid cycle (Patel et al., 2012; Hussein and Alzamily, 2024). Riboflavin is transported into the cytoplasm and degenerates to form dimethylbenzimidazole and flavin mononucleotides, resulting in the formation of flavin adenine dinucleotide (FAD) in the mitochondria. In melanosomes, quinone generates hydroquinone through the catalysis of 6,7-dimethyl-8-(1-D-ribityl) lumazine originating from intestinal microflora to form 7-hydroxy-6-methyl-8-ribityllumazine. These reaction products can be recycled with the aid of probiotic bacteria to synthesize recyclable riboflavin in *B. subtilis* cells (Maslowski, 2019).

These metabolites secreted by *B. subtilis YB-1*14246 effectively optimize the bacterial abundances in the ileum and cecum of the chicken. At the phylum level, the *Firmicutes*-to-Bacteroides ratio increased (Yang et al., 2020). An increased proportion of *Firmicutes* can enhance nutrient absorption. In addition, the abundance of the functional genera *Ruminococcaceae*_ UCG-005 and g_ unclassified _f_ *Ruminococcaceae* improved. Some functional metabolites can be transported through the blood to assist other organs in their respective roles. Non-ribosomal peptides and lantipeptides can improve the antioxidant and antimicrobial roles of the liver and heart (Alippi et al., 2023). In addition, intestinal immunity can be improved (Yang et al., 2021).

Metabolites identified *in vivo* cannot be fully absorbed into the bodies of hens; therefore, the riboflavin concentration that reached the cells of the organs was unknown. Direct evaluation of probiotic bacteria residing in simulated organs has been lacking; therefore, our study provides valuable insights. The correlations between laying performance and metabolites were based on only one or two components, which were also not fully covered, thereby affecting subsequent studies. However, innovation based on the detected techniques and statistical analyses can help to further advance our understanding.

Overall, valuable insights into the genes and metabolic pathways of *B. subtilis YB-1*14246 can help us address the physiological shortcomings of animals and optimally select probiotic bacteria. However, practical probiotic applications often involve the combined use of multiple strains rather than a single one. Future studies should therefore focus on determining optimal proportions of two or more probiotic strains and evaluating their synergistic effects when supplemented.

## Data Availability

The data of whole gene sequence and metabonosim were deposited in NCBI, the accession number is PRJNA1142186, SAMN 17073208: *Bacillus subtilis yb-1*14246 (TaxID: 1423), SAMN42929163: *Bacillus subtilis* metabonomes (TaxID: 1239). *B*. *subtilis* CPS52 (NCBI: LZOV01000014.1, https://www.ncbi.nlm.nih.gov/nuccore/LZOV01000014.1) and *B. subtilis* AJQ03 (NCBI:NZ_JABFHE010000001.1, https://www.ncbi.nlm.nih.gov/nuccore/NZ_JABFHE010000001.1).

## Footnotes

^1^ http://support.illumina.com/sequencing/sequencing_software/casava.ilmn

^2^ http://www.usadellab.org/cms/uploads/supplementary/Trimmomatic

^3^ http://uniprot.org

^4^ http://www.genome.jp/kegg/

^5^ http://www.ncbi.nlm.nih.gov/COG

^6^ https://github.com/bbuchfink/diamond

^7^ http://ftp.ncbi.nlm.nih.gov/blast/executables/blast+/2.3.0/

^8^ http://eggnogdb.embl.de/#/app/home

^9^ https://dl.secondarymetabolites.org/releases/4.0.2/
