# Supplementary material for: Characterizing probiotic profiles using genomic and metabolomic insights into chicken cecum-derived Bacillus subtilis YB-114246
Source: Front Microbiol. 2025 Nov 13;16:1706551. doi: 10.3389/fmicb.2025.1706551 (PMC12659908; doi:10.3389/fmicb.2025.1706551)
Supplement: Supplementary file 1 [file Data_Sheet_1.pdf]

Supplementary Table 1 Information on identified functional genes by KEGG category, main metabolic compounds secreted by *B. subtilis yb-1*14246.

Identified functional genes and numbers		Major metabolic compounds and Concentration μg/L N=12		
Metabolism	917	Groups	Fermentation	Control
carbohydrate metabolic	319	Riboflavin	6.76A	2.53B
glycan	104	P-acetaminobenzoic acid	6.65	6.05
amino acids	109	6-Hydroxyhexanoic acid	7.03	6.47
nucleotide and lipid	63	3-Phenyllatic acid	1.67	1.59
Vitamin, short peptides, and cholesterol	48	deca-2,5,8-trienedioylcarnitine	5.09	3.93
		SE	0.33	

Note: *B. subtilis yb-1*14246 was cultured in YEPD medium for 18h. The cell of *B. subtilis yb-1*14246 was collected and extracted the total DNA, and be whole sequenced to predict its functional genes and their number. The fermentation liquid was also sampled to detect the main metabolic compounds and digestive enzymes fit for laying performance. SE: standardized error.

Supplementary Table 2 The overview of total genes owning by three strains of *Bacillus subtilis yb-1*14246.

Samples Name	yb114246	CPS52	AJQ03
Total gene	3798	369	352
core	335	331	331
unique	3463	38	21

Supplementary Table 3 The comparison on secondary metabolites synthesized by the clusters of three strains of *B. subtilis yb-1*14246.

Cluster Type	*B. subtilis yb1*14246	*B. subtilis* AJQ03	*B. subtilis* CPS52
Non-ribosomal peptides	1	1	1
Lantipeptide	1	1	0
Terpene	2	0	0
Trans AT PKS	3	0	0
Type III PKS	1	0	0
Bacteriocin	2	0	0

Supplementary Table 4 The evaluative values on the predictive capacity of the model

Components	R2X	R2X (cum)	R2Y	R2Y (cum)	Q2	Q2 (cum)
C1	0.793	0.793	0.505	0.505	0.502	0.502
C2	0.0522	0.846	0.478	0.983	0.923	0.962
C3	0.0125	0.858	0.0147	0.988	0.463	0.979
